# Effectiveness of advanced dressings in preventing surgical site infections compared to that of standard dressings in gastrointestinal surgery: A systematic review and meta‐analysis for guideline revision by the Japanese Society for Surgical Infection

**DOI:** 10.1002/ags3.12909

**Published:** 2025-01-08

**Authors:** Keita Kouzu, Hironori Tsujimoto, Seiichi Shinji, Hiroji Shinkawa, Koji Tamura, Yukio Sato, Koji Munakata, Yasunari Fukuda, Daisuke Koike, Hiromu Miyake, Yohei Hosoda, Motoi Uchino, Hiroki Ohge, Junzo Shimizu, Seiji Haji, Yasuhiko Mohri, Chizuru Yamashita, Yuichi Kitagawa, Motomu Kobayashi, Yuki Hanai, Hiroshi Nobuhara, Masahiro Yoshida, Toru Mizuguchi, Toshihiko Mayumi, Yuko Kitagawa

**Affiliations:** ^1^ Department of Surgery National Defense Medical College Saitama Japan; ^2^ Department of Gastroenterological Surgery Nippon Medical School Japan; ^3^ Department of Hepatobiliary‐Pancreatic Surgery Osaka Metropolitan University Graduate School of Medicine Japan; ^4^ Department of Surgery and Oncology, Graduate School of Medical Sciences Kyushu University Fukuoka Japan; ^5^ Department of Emergency and Critical Care Medicine Keio University School of Medicine Japan; ^6^ Department of Gastroenterological Surgery Ikeda City Hospital Osaka Japan; ^7^ Department of Gastroenterological Surgery Kindai University Nara Hospital Nara Japan; ^8^ Department of Gastroenterological Surgery Fujita Health University Bantane Hospital Aichi Japan; ^9^ Department of Pediatric Surgery Shizuoka Children's Hospital Shizuoka Japan; ^10^ Department of Surgery Tane General Hospital Osaka Japan; ^11^ Department of Gastroenterological Surgery, Division of Inflammatory Bowel Disease Hyogo Medical University Hyogo Japan; ^12^ Department of Infectious Diseases Hiroshima University Hospital Hiroshima Japan; ^13^ Department of Surgery Toyonaka Municipal Hospital Osaka Japan; ^14^ Department of Surgery Soseikai General Hospital Kyoto Japan; ^15^ Department of Surgery Mie Prefectural General Medical Center Mie Japan; ^16^ Department of Anesthesiology and Critical Care Medicine Fujita Health University School of Medicine Aichi Japan; ^17^ Department of Infection Control National Center for Geriatrics and Gerontology Aichi Japan; ^18^ Department of Anesthesiology Hokushinkai Megumino Hospital Hokkaido Japan; ^19^ Department of Clinical Pharmacy, Faculty of Pharmaceutical Sciences Toho University Chiba Japan; ^20^ Department of Dentistry and Oral and Maxillofacial Surgery Hiroshima Prefectural Hospital Hiroshima Japan; ^21^ Department of Hepato‐Biliary‐Pancreatic and Gastrointestinal Surgery International University of Health and Welfare, School of Medicine Chiba Japan; ^22^ Department of Nursing, Division of Surgical Science Sapporo Medical University Hokkaido Japan; ^23^ Department of Intensive Care Unit Japan Community Healthcare Organization Chukyo Hospital Aichi Japan; ^24^ Department of Surgery Keio University School of Medicine Japan

**Keywords:** gastrointestinal surgery, meta‐analysis, surgical site infection, systematic review, wound dressings

## Abstract

**Introduction:**

This is a systematic review and meta‐analysis of the efficacy of wound coverage using advanced dressings specifically for the prevention of surgical site infections (SSI) in gastrointestinal surgery, as part of the update of the SSI prevention guidelines of the Japan Society for Surgical Infection (JSSI).

**Methods:**

After searching CENTRAL, PubMed, and ICHUSHI‐Web in July 2024, we included randomized controlled trials (RCTs) comparing advanced dressings and standard dressings for surgical wounds in gastrointestinal surgery (PROSPERO No. CRD42024569084). Three authors independently screened the RCTs. We assessed the risk of bias and certainty of the body of evidence for the extracted data. The primary outcome was superficial SSI, and the secondary outcomes were length of postoperative hospital stay, costs, and allergy. This study was partially supported by the JSSI.

**Results:**

A total of seven RCTs and 927 patients were included. The use of advanced dressings significantly lowered the risk of SSI compared to that associated with standard dressings (risk ratio: 0.54, 95% confidence intervals: 0.34–0.88). The certainty of the evidence was rated as moderate. According to the subgroup analysis, advanced dressings reduced the risk of SSI in colorectal surgery. Advanced dressings did not reduce the length of postoperative hospital stay or costs compared to that of standard dressings. Allergies were reported in only one patient using silver‐impregnated dressings.

**Conclusion:**

The use of advanced dressings for primary wounds in gastrointestinal surgery was associated with a significantly lower risk of SSI than that associated with standard dressings.

## Graphical abstract

This is a systematic review and meta‐analysis of the efficacy of wound coverage using advanced dressings specifically for the prevention of surgical site infections (SSI) in gastrointestinal surgery. We found that the use of advanced dressings for primary wounds in gastrointestinal surgery was associated with a significantly lower risk of SSI than that associated with standard dressings.

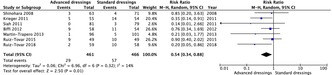

## 1 INTRODUCTION

Surgical‐site infections (SSI) are among the most common postoperative complications. They cause symptoms such as fever and pain and also impact hospitalization duration, aesthetics, and medical costs.^1^ Therefore, healthcare professionals involved in surgery must implement measures to minimize the incidence of SSI.

There are numerous strategies for preventing SSI, which can be categorized into preoperative, intraoperative, and postoperative measures.^2^ One postoperative measure to prevent incisional SSI is wound management. Surgically created and closed wounds epithelialize within approximately 48 h through the wound healing process, and during this period, surgical wounds are generally covered with dressings. Dressings serve as a physical barrier that protects the wound from external contamination and absorb exudates from the wound. Traditionally, gauze has been used to cover wounds; however, it is insufficient for preventing SSI as it allows the passage of bacteria. Dressings that create a closed environment and maintain an appropriate moist environment are recommended to promote wound healing and reduce the incidence of SSI.^3^

Regarding the use of dressings for the prevention of SSI in surgical wounds, the Centers for Disease Control and Prevention (CDC) states that there are uncertain trade‐offs between the benefits and harms of antimicrobial dressings applied to surgical incisions after primary closure in the operating room for the prevention of SSI (no recommendation/unresolved issue).^4^ In contrast, the World Health Organization (WHO) suggests not using any type of advanced dressing over a standard dressing on primarily closed surgical wounds to prevent SSI (conditional recommendation/low quality of evidence).^2^ Thus, there is no consistent view on the effectiveness of dressings in preventing SSI.

These guidelines and the representative Cochrane review on wound dressings encompassed cases across various fields, including cardiovascular surgery, gastrointestinal surgery, gynecology, and orthopedic surgery.^5^ In gastrointestinal surgery, where the incidence of SSI is high, the prevention of SSI is even more critical. However, no clear recommendations exist specifically for gastrointestinal surgery. The Japan Society for Surgical Infection (JSSI) conducted a systematic review and meta‐analysis in 2018 from this perspective and recommended in their guidelines to place protective wound dressings over large incisional wounds after abdominal surgery, rather than covering them with gauze.^6^ The JSSI guidelines include randomized controlled trials (RCTs) from the 1980s, and the contents of which may be obsolete. Additionally, the guidelines must always be updated with new data, necessitating a search for new findings from previous editions. Therefore, we aimed to reanalyze the effectiveness of advanced dressings in preventing SSI in the field of gastrointestinal surgery to update the JSSI guidelines.

## 2 METHODS

### 2.1 Registration and protocol

This systematic review and meta‐analysis was registered in advance in PROSPERO (http://www.crd.york.ac.uk/PROSPERO; CRD42024569084). The study procedures were in accordance with the Preferred Reporting Items for Systematic Reviews and Meta‐Analyses (PRISMA) guidelines (Supplementary Material S1). Ethical approval was not required due to the study design.

### 2.2 Eligibility criteria

The predefined inclusion criteria for this systematic review were as follows: (1) RCTs published between January 2000 and December 2023; (2) studies targeting primary incisions in gastrointestinal surgeries classified as clean‐contaminated or contaminated; (3) studies involving cases with standard dressings for wound protection as the control group; and, (4) studies involving cases with advanced dressings or silver‐impregnated dressings for wound protection as the intervention group. The definitions of standard dressings, advanced dressings, and silver‐impregnated dressings were based on the WHO Surgical Site Infection Prevention Guidelines, Appendix 26: Summary of the systematic review on advanced dressings.^2^ The exclusion criteria were as follows: (1) studies targeting only clean, dirty, or infected incisions; (2) studies focusing solely on emergency surgeries; (3) studies targeting only secondary incisions; and, (4) studies using negative pressure wound therapy (NPWT). Studies that examined stoma wound closure were excluded. There were no restrictions based on country, age, sex, surgical approach (open surgery, laparoscopic surgery, or robot‐assisted surgery), or sample size.

### 2.3 Information sources and search strategy

We searched the Cochrane Central Register of Controlled Trials (CENTRAL) in Cochrane Library, MEDLINE (PubMed), and ICHUSHI Web (the Japanese search engine of the Japan Medical Abstracts Society) for articles published between January 2000 and December 2023. The search strategy is described in detail in Supplementary Material S2. Additionally, we conducted a manual search for relevant papers. The final search date was July 28, 2024.

### 2.4 Selection process

For all papers extracted using the above search strategy, three reviewers (S.S., K.K., and T.M.) independently conducted primary screening based on the titles and abstracts using Rayyan (https://www.rayyan.ai). Further, the same three reviewers independently conducted secondary screening of the full texts of the papers selected during the primary screening. Disagreements between the reviewers regarding the inclusion of specific papers were resolved through discussion.

### 2.5 Outcomes and data items

The primary outcome was superficial SSI (hereinafter, referred to as SSI) within 30 postoperative days. Secondary outcomes included duration of postoperative hospital stay, costs, and allergies to the dressing components. From the studies meeting the inclusion criteria, the following information was independently extracted using a preset standard form: first author, publication year, country, study design, single‐center or multicenter study, type of surgery, wound class according to the CDC classification,^4^ number of participants, event numbers for binary data, mean and standard deviation (SD) for continuous data, and funding sources. These data items were extracted solely from the information reported in published data.

### 2.6 Risk of bias assessment

The risk of bias in the included studies was assessed using criteria described in the Cochrane Handbook for Systematic Reviews of Interventions.^7^

### 2.7 Synthesis method

The meta‐analysis was conducted using the Review Manager (RevMan, Version 5.3; Copenhagen: The Nordic Cochrane Centre, The Cochrane Collaboration, 2014). A random‐effects model was used to assess intergroup differences. These differences were quantified as risk ratios (RR) with 95% confidence intervals (CI) for binary data and as mean differences (MD) with 95% CI for continuous data. Heterogeneity was initially evaluated by a visual inspection of forest plots. The degree of heterogeneity was quantified using the *I* ^*2*^ statistic, and Tau^2^, representing the between‐study variance in the random‐effects model, was also reported. The funnel plot was generated using EZR (version 1.61, Saitama Medical Center, Jichi Medical University, Saitama, Japan).

### 2.8 Reporting bias assessment

Publication bias was assessed using Egger test and a visual inspection of funnel plots.

### 2.9 Certainty assessment

Overall certainty in the body of evidence was determined by voting among the authors based on criteria such as the study design, risk of bias, inconsistency, imprecision, indirectness, and publication bias.

## 3 RESULTS

### 3.1 Study selection

**FIGURE 1:**
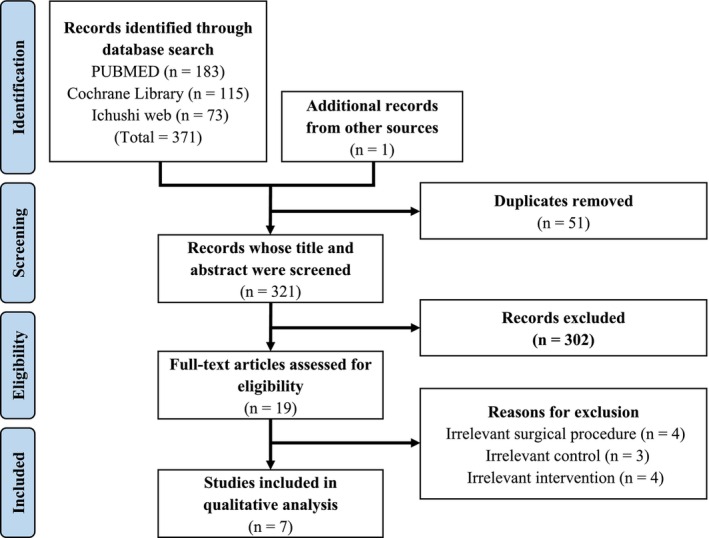
Flow diagram illustrating the process from the search for studies to the extraction of studies included in the meta‐analysis. Ultimately, seven RCTs were analyzed.

A total of 372 articles were identified. After excluding duplicate articles and conducting primary and secondary screening, seven RCTs were included.^8^, ^9^, ^10^, ^11^, ^12^, ^13^, ^14^ This process is illustrated in Figure 1. The included articles are listed in Table 1. Ruiz‐Tovar et al. compared patients in three groups: silver‐impregnated dressings, mupirocin ointments, and standard dressings.^13^ Data of the groups with silver‐impregnated and standard dressings were extracted. Based on the results of the systematic review, two studies reported by same authors, Ruiz‐Tovar et al., were extracted. However, as the cases targeted by these RCTs were entirely different, both studies were included in the meta‐analysis. The study by Arroyo et al. was excluded because approximately 70% of the surgeries were orthopedic.^15^ Additionally, the study by Ezzelarab et al. was excluded because 88% of the included surgical wounds were categorized as clean.^16^

**TABLE 1:** Characteristics of included studies.

Author	Year	Country	Study design	Surgical timing	Type of surgery	Open/laparoscopy	SSI definition	Intervention	Control
Shinohara^8^	2008	Japan	Single	Elective	Gastrointestinal	N/A	CDC	Hydrocolloid dressing	Gauze dressing
Krieger^9^	2011	USA	Single	Elective	Colorectal	Mixed	CDC	Silver nylon dressing	Gauze dressing
Siah^10^	2011	Singapore	Single	Elective	Colorectal	Mixed	CDC	Ionic silver‐containing dressing	Sterile, highly absorbent, low‐adherent pad
Biffi^11^	2012	Italy	Multicenter	Elective	Colorectal	Laparoscopy	CDC	Silver‐containing hydrofiber	Standard absorbent dressing
Martin‐Trapero^12^	2013	Spain	Single	Elective	Cholecystectomy	Laparoscopy	CDC	Polyhexamethylene biguanide dressing	Gauze dressing
Ruiz‐Tovar^13^	2015	Spain	Single	Elective	Colorectal	Open	CDC	Silver‐containing hydrofiber	Gauze dressing
Ruiz‐Tovar^14^	2018	Spain	Multicenter	Elective	Colorectal	Laparoscopy	CDC	Vitamin E‐silicone containing dressing	Gauze dressing

Abbreviations: CDC, the Centers for Disease Control and Prevention; N/A, Not available; SSI, Surgical site infection.

### 3.2 Study characteristics

All seven included RCTs used standard dressings as controls; however, the dressings used in the interventions varied (Table 1). Four RCTs used silver‐impregnated dressings as an intervention,^9^, ^10^, ^11^, ^13^ and one RCT investigated hydrocolloid dressings,^8^ polyhexametylene biguanide dressings,^12^ and Vitamin E‐containing silicone dressings.^14^ Five RCTs focused on colorectal surgery;^9^, ^10^, ^11^, ^13^, ^14^ one, general gastrointestinal surgery;^8^ and, one, cholecystectomy.^12^ Regarding surgical approaches, one RCT involved open surgery;^13^ three, laparoscopic surgery;^11^, ^12^, ^14^ two, a combination of approaches;^9^, ^10^ and, one, did not specify.^8^ None of the RCTs included robot‐assisted surgeries. Although no RCTs reported clear conflicts of interest, one RCT received dressing materials from a company.^14^

### 3.3 Risk of bias in studies

**FIGURE 2:**
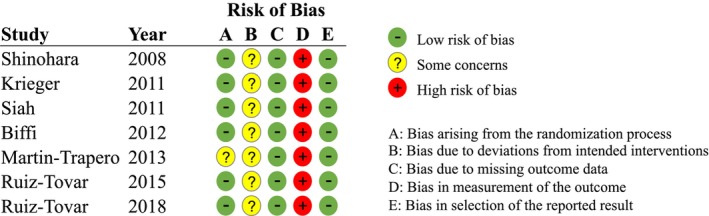
Risk of bias assessment results for the studies included in this analysis. Due to the nature of the study evaluating wound dressings, bias in the blinding process was unavoidable.

The results of the risk of bias analysis are shown in Figure 2. Due to the nature of the study, it was challenging to fully blind patients and healthcare providers to the allocation of patients to either the control or intervention groups. However, intentional deviations from the intended treatment due to insufficient blinding were not evident; thus, the bias due to deviations from intended interventions was judged as “some concern” in all RCTs. Additionally, achieving complete blinding for the SSI evaluators was difficult, and the possibility that this influenced the SSI assessment could not be ruled out. Therefore, the bias in the measurement of the outcome was judged as “high risk of bias” in all RCTs. Furthermore, one study could not rule out baseline imbalances, leading to the judgment of “some concern” for biases arising from the randomization process.^12^

### 3.4 Results of individual studies

#### 3.4.1 Surgical site infections in all advanced dressings

**FIGURE 3:**
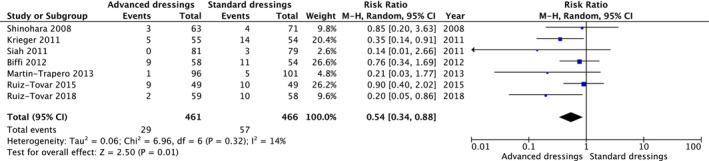
Forest plot of the meta‐analysis comparing the incidence of SSI between any advanced dressings and standard dressings. The use of advanced dressings significantly reduced the incidence of SSI compared to the use of standard dressings. CI, confidence interval; M‐H, Mantel–Haenszel test; SSI, surgical site infection.

A meta‐analysis was conducted on all seven included RCTs to assess the impact of advanced dressings on the incidence of SSI compared to standard dressings (Figure 3). A total of 466 patients were enrolled in the standard dressing group, and 461 patients were enrolled in the advanced dressing group, with 57 and 29 events occurring in each group, respectively. The incidence of SSI was significantly lower with the use of advanced dressings than that with the use of standard dressings [RR: 0.54, 95% CI: 0.34–0.88]. Two RCTs demonstrated that advanced dressings significantly reduced the incidence of SSI compared to that in standard dressings. Five RCTs suggested the preventive effect of advanced dressings on SSI, although the difference was not statistically significant. In summary, the results of all seven RCTs either favored the intervention or showed a tendency towards it. The heterogeneity was judged to be low (*I* ^*2*^ = 14%, Tau^2^ = 0.06). The combined effect was statistically significant (*p* = 0.01). The body of evidence is presented in Table 2. Owing to the nature of the studies, the blinding of patients, healthcare providers, and outcome assessors was challenging, leading to a very serious risk of bias. Inconsistencies were judged to be serious because of variations in interventions. Publication bias was recognized owing to the limited reporting of negative studies (Figure S1a).

**TABLE 2:** The body of evidence.

Quality assessment										Effect		Quality	Importance
Comparisons	Outcomes	Study design/studies	Risk of bias	Inconsistency	Imprecision	Indirectness	Other considerations	Intervention	Control	Relative (95%CI)	Absolute (95%CI)		
Advanced versus standard dressings (any type of surgery)	SSI	RCT/7	Very serious	Not serious	Not serious	Very serious	Reporting bias	29/461 (6.3%)	57/466 (12.2%)	RR: 0.54 (0.34–0.88)	56 fewer per 1000 (81 fewer to 15 fewer)	Moderate	Critical
Advanced versus standard dressings (colorectal surgery)	SSI	RCT/5	Very serious	Not serious	Not serious	Not serious	Reporting bias	25/302 (8.3%)	48/294 (16.3%)	RR: 0.52 (0.29–0.94)	76 fewer per 1000 (116 fewer to 10 fewer)	High	Critical
Silver‐impregnated versus standard dressings	SSI	RCT/4	Very serious	Not serious	Not serious	Not serious	Reporting bias	23/243 (9.5%)	38/236 (16.1%)	RR: 0.62 (0.36–1.06)	61 fewer per 1000 (103 fewer to 10 more)	High	Critical

#### 3.4.2 Surgical site infections in silver‐impregnated dressings

**FIGURE 4:**
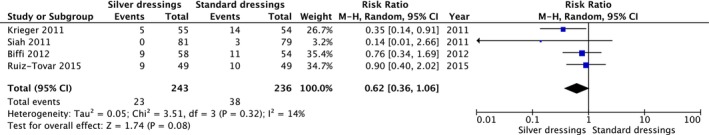
Forest plot of the meta‐analysis comparing the incidence of SSI between silver‐impregnated dressings and standard dressings. The use of silver‐impregnated dressings did not show a statistically significant reduction in SSI incidence compared to that of standard dressings. CI, confidence interval; M‐H, Mantel–Haenszel test; SSI, surgical site infection.

When the dressings used in the intervention were limited to silver‐impregnated dressings, no impact on the incidence of SSI was observed compared to that in the standard dressings [RR: 0.62, 95% CI: 0.36–1.06] (Figure 4). The heterogeneity in this meta‐analysis might not be important (*I* ^*2*^ = 14%, Tau^2^ = 0.05). The combined effect was not statistically significant (*p* = 0.08). Publication bias was recognized due to the limited reporting of negative studies (Figure S1b).

#### 3.4.3 Surgical site infections in colorectal surgery

**FIGURE 5:**
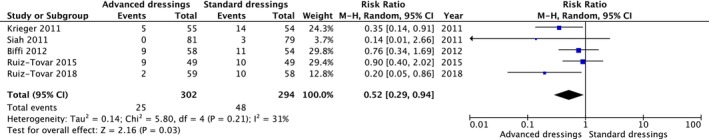
Forest plot of the meta‐analysis comparing the incidence of SSI between any advanced dressings and standard dressings, specifically limited to colorectal surgery. The use of advanced dressings significantly reduced the incidence of SSI compared to the use of standard dressings. CI, confidence interval; M‐H, Mantel–Haenszel test; SSI, surgical site infection.

Five of the RCTs targeted colorectal surgery. When limited to these RCTs, a statistically lower incidence of SSI was observed with advanced dressings comparing to that with standard dressings [RR: 0.52, 95% CI: 0.29–0.94] (Figure 5). The heterogeneity of this meta‐analysis was determined to be *I* ^*2*^ = 31% and tau^2^ = 0.14, indicating that heterogeneity might not be important. The pooled effect showed a statistically significant difference with *p* = 0.03. Publication bias was noted due to the scarcity of negative study reports (Figure S1c).

#### 3.4.4 Length of hospital stay

The length of hospital stay was examined in only one RCT comparing silver‐impregnated dressings with standard dressings.^10^ In the RCT, the mean hospital stay was 8.5 ± 6.9 days in the standard dressing group, compared to 9.4 ± 4.5 days in the silver‐impregnated dressings group. No significant improvement in the length of hospital stay was observed with the intervention of silver‐impregnated dressings [MD, −0.90; 95% CI: −2.67–0.87]. Since there was only one RCT, a meta‐analysis was not conducted.

#### 3.4.5 Costs

Only one RCT examined the cost, comparing hydrocolloid dressings with standard dressings.^8^ The cost calculation in the RCT considered the frequency of dressing changes required for each treatment group, cost per dressing, and cost of povidone‐iodine and cotton balls used during dressing changes. The mean cost was 779.9 ± 345.3 Japanese yen in the standard dressing group, compared to 714.9 ± 262.8 yen in the hydrocolloid group, with no significant difference observed between the two groups [MD: −65.00, 95% CI: −168.26–38.26]. Due to the availability of only a single RCT, a meta‐analysis was not performed.

#### 3.4.6 Allergy

Allergies were reported in only one patient who used silver‐impregnated dressings.^9^ The patient developed a rash at the dressing site that resolved promptly upon discontinuation of the dressing. Due to the small number of events, a meta‐analysis was not conducted.

## 4 DISCUSSION

This study is a systematic review and meta‐analysis of the incidence of SSI associated with different dressings used for primary incisions in gastrointestinal surgery. The results from seven RCTs demonstrated that advanced dressings significantly reduced SSI compared to standard dressings. In particular, five RCTs indicated that advanced dressings were beneficial in colorectal surgery. Conversely, silver‐impregnated dressings did not significantly reduce SSI as much as standard dressings did. Only one RCT addressed the length of hospital stay and costs, showing no significant reduction in SSI with advanced dressings compared to that of standard dressings. Allergic reactions to dressings were mentioned in one RCT concerning silver‐impregnated dressings, where only one of 109 cases exhibited a local allergic reaction that improved upon dressing removal. Based on these findings, we weakly recommend the use of advanced dressings for the postoperative wound coverage of primary incisions in gastrointestinal surgery.

One RCT included in this study reported no significant cost differences between hydrocolloid and standard dressings.^8^ However, in this study, dressings were used until postoperative day 7 unless a clinical wound infection developed. The frequency of dressing changes was significantly higher in the standard dressing group (11.8 ± 6.0 times) compared to that in the hydrocolloid group (2.1 ± 0.8 times), and povidone‐iodine was used in wound management. Therefore, if routine practice at a facility involves earlier postoperative dressing removal than that in this trial, standard dressings, which are less expensive, may be more cost‐effective because of the reduced need for frequent dressing changes. However, the costs associated with SSI occurrence were not analyzed in the RCT, and the impact of hydrocolloid intervention on costs throughout the postoperative period is unclear. There are several potential explanations for why advanced dressings did not reduce costs despite their effectiveness in reducing SSI. First, one possibility is that the SSIs identified in this systematic review were superficial SSIs that could be managed on an outpatient basis, and thus did not lead to prolonged postoperative hospital stays. Second, only one RCT was available for each of the outcomes related to the length of hospital stay and costs,^8^, ^10^ which may have resulted in a lack of statistical impact. Finally, since these RCTs did not demonstrate a statistically significant reduction in SSI with the use of advanced dressings, it is possible that there was no effect on the length of hospital stay or costs either.

This analysis has several limitations. First, the number of RCTs was limited to seven, each with a small sample size, resulting in a total of just 927 cases. Additionally, publication bias was present, imposing further constraints on the analysis. Second, owing to the nature of the clinical question, blinding was challenging in these trials, resulting in a low certainty of the evidence. Furthermore, there were inconsistencies in the materials used as dressings for the interventions.

There are limitations to this review process. One limitation was the potential changes in basic SSI prevention measures over time other than dressings. For instance, the duration of postoperative dressing in the RCTs included in this review varied widely, ranging from 48 h^12^ to 5 days,^13^, ^14^ 7 days,^8^, ^10^, ^11^ and 14 days.^9^ It cannot be denied that there may be inherent variability in postoperative wound management practices due to differences in time periods, countries, and institutions. Furthermore, the potential lack of uniformity in surgical techniques and postoperative management between the control and intervention groups should be considered. For instance, in gastrointestinal surgery, subcuticular closure with absorbable sutures reduced the incidence of incisional SSI compared to closure with other skin suturing devices such as skin staplers or non‐absorbable sutures.^17^ In addition, there are technical differences between continuous and interrupted sutures used for skin closure. Therefore, RCTs where all surgical techniques related to skin closure are standardized must be designed.

In the RCTs excluded from this study because the majority of the performed surgeries were clean surgeries, there was no difference in the incidence of SSI between the group using standard dressings and the group exposed to the environment without wound protection materials.^18^ This suggests that the necessity of covering wounds for SSI prevention should be reconsidered depending on the surgical procedure. Regarding the surgical procedures, although an integrated analysis was possible for colorectal surgery in this study, subgroup analyses of other areas were not feasible. Therefore, it is important to recognize that the results of this meta‐analysis are primarily based on data from colorectal surgery. Furthermore, evidence specific to minimally invasive surgeries, including the increasingly common robot‐assisted surgeries, was limited.

Kosugi et al. reported the results of an RCT comparing film dressings and silver‐impregnated dressings in patients who underwent gastrointestinal surgery.^19^ Although this study was not included in our review because standard dressings were not used as the control (film dressings are classified as advanced dressings), it has been reported that silver‐impregnated dressings can reduce superficial SSIs compared with film dressings in patients undergoing elective gastrointestinal surgery, particularly in lower gastrointestinal procedures. As the use of advanced dressings has become more common for covering primary incisions following gastrointestinal surgery, a clinical question has arisen regarding the specific dressings that should be employed. Future RCTs should aim to identify appropriate advanced dressings. Previous network meta‐analyses that attempted to identify the most effective dressings for reducing SSI were not conclusive because of overly detailed classifications of dressings and the inclusion of surgeries beyond gastrointestinal surgery, failing to address our clinical question.^20^ More RCTs are required to conduct a network meta‐analysis. Moreover, as mentioned above, our meta‐analysis encompasses multiple areas within the field of gastrointestinal surgery and includes various materials classified as advanced dressings, thus requiring careful interpretation of the results in light of these factors.

In addition, this meta‐analysis and systematic review did not include NPWT, which is an aggressive postoperative wound management approach. Negative pressure wound therapy involves the application of a sealed system connected to a vacuum pump to maintain negative pressure at the wound, thereby promoting wound healing and potentially reducing SSI.^21^ However, considering the cost, management complexity, and patient burden, the routine use of NPWT for primary incisions is an optional practice.^22^ However, NPWT has been suggested to be beneficial in abdominal surgery,^2^ and high‐quality evidence is needed to identify patient populations that could benefit from NPWT. It is desirable that future systematic reviews and meta‐analyses on the prevention of SSI with wound protectants include NPWT in addition to dressings.

Finally, the initial version of the JSSI guidelines also recommends the use of advanced dressings, and it is anticipated that the frequency of standard dressings in clinical practice in Japan is decreasing. However, we did not have specific data to present regarding the current utilization of these dressings. In this regard, we recognize the need to conduct future surveillance to clarify the significance of addressing this theme within the guidelines.

In conclusion, although the disadvantages of using advanced dressings are acceptable and there are inconsistencies in dressing materials, the use of any advanced dressing was associated with a potential reduction in SSI compared with that associated with standard dressings. Therefore, we weakly recommend the use of advanced dressings for the postoperative wound coverage of primary incisions during gastrointestinal surgery. Advanced dressings should be utilized as part of an SSI prevention bundle, with a clear understanding of the limitations inherent in this study. Future studies should involve high‐quality RCTs with large sample sizes, standardized surgical procedures, and skin closure techniques.

## AUTHOR CONTRIBUTIONS

**Keita Kouzu:** Conceptualization; data curation; formal analysis; investigation; methodology; software; visualization; writing – original draft. **Hironori Tsujimoto:** Writing – review and editing. **Seiichi Shinji:** Conceptualization; data curation; formal analysis; investigation. **Hiroji Shinkawa:** Investigation. **Koji Tamura:** Investigation. **Yukio Sato:** Investigation. **Koji Munakata:** Investigation. **Yasunari Fukuda:** Investigation. **Daisuke Koike:** Investigation. **Hiromu Miyake:** Investigation. **Yohei Hosoda:** Investigation. **Motoi Uchino:** Investigation. **Hiroki Ohge:** Investigation. **Junzo Shimizu:** Investigation. **Seiji Haji:** Investigation. **Yasuhiko Mohri:** Investigation. **Chizuru Yamashita:** Investigation. **Yuichi Kitagawa:** Investigation. **Motomu Kobayashi:** Investigation. **Yuki Hanai:** Investigation. **Hiroshi Nobuhara:** Investigation. **Masahiro Yoshida:** Methodology; project administration; supervision. **Toru Mizuguchi:** Conceptualization; data curation; formal analysis; funding acquisition; investigation; methodology; project administration; resources; validation. **Toshihiko Mayumi:** Project administration; supervision. **Yuko Kitagawa:** Funding acquisition; project administration.

## FUNDING INFORMATION

This study was supported in part by the Japan Society for Surgical Infection.

## CONFLICT OF INTEREST STATEMENT

Y.K. reports grants from Asahi Kasei Pharma Corporation, grants from Ono Pharmaceutical Co., Ltd., grants from Otsuka Pharmaceutical Factory Inc., grants from Nippon Covidien Inc., grants from Taiho Pharmaceutical Co., Ltd., grants from Chugai Pharmaceutical Co., Ltd., grants from Kaken Pharmaceutical Co., Ltd., grants from EA Pharma Co., Ltd., grants from Yakult Honsha Co., Ltd., grants from Otsuka Pharmaceutical Co., Ltd., grants from Tsumura & CO., grants from Sumitomo Pharma Co., Ltd., grants from Eisai Co., Ltd., grants from Kyouwa Kirin Co., Ltd., grants from Takeda Pharmaceutical Co., Ltd., grants from Teijin Pharma Limited., grants from Cardinal Health., grants from Kowa Company, Ltd., personal fees from Asahi Kasei Pharma Corporation, personal fees from AstraZeneca K.K., personal fees from Ethicon Inc., personal fees from Ono Pharmaceutical Co., Ltd., personal fees from Otsuka Pharmaceutical Factory, Inc., personal fees from Olympus Corporation, personal fees from Cardinal Health. K.K., personal fees from Shionogi & Co., Ltd., personal fees from Taiho Pharmaceutical Co., Ltd., personal fees from Chugai Pharmaceutical Co., Ltd., personal fees from Bristol‐Myers Squibb K.K., personal fees from MSD K.K., personal fees from Smith & Nephew KK, personal fees from Kaken Pharmaceutical Co., Ltd., personal fees from ASKA Pharmaceutical Co., Ltd. personal fees from Miyarisan Pharmaceutical Co., LTD., personal fees from Toray Industries, Inc., personal fees from Daiichi Sankyo Company, Ltd., personal fees from Chugai Foundation for Innovative Drug Discovery Science, personal fees from Nippon Kayaku Co., Ltd., personal fees from EA pharma Co., Ltd., personal fees from Intuitive Surgical G.K., personal fees from Takeda Pharmaceutical Company Ltd., personal fees from Sysmex Corporation, and personal fees from Tsumura & Co. outside the submitted work. The remaining authors have no conflicts of interest. H.T. and H.O. are editorial board members of *Annals of Gastroenterological Surgery*. Y.K. is an Editor‐in‐Chief of *Annals of Gastroenterological Surgery*.

## ETHICS STATEMENT

Ethical approval was not required due to the study design.

Approval of the research protocol by an Institutional Reviewer Board: N/A.

Informed Consent: N/A.

Registry and the Registration No. of the study/trial: N/A.

Animal Studies: N/A.

## Supporting information


**Figure S1:** The symmetrical funnel plots of meta‐analysis (a) comparing the incidence of surgical site infection (SSI) between any advanced dressings and standard dressings, (b) comparing the incidence of SSI between silver‐impregnated dressings and standard dressings, (c) comparing the incidence of SSI between any advanced dressings and standard dressings, specifically limited to colorectal surgery. All funnel plots suggest reporting bias with a paucity of studies with negative results.

**Data S1:** Supplementary Material 1: PRISMA 2020 Checklist.

**Data S2:** Supplementary Material 2: Search strategy in Medline (through PubMed).

## Data Availability

All data used in this manuscript are available on request.
