# Supplementary figures and images for: Effectiveness of advanced dressings in preventing surgical site infections compared to that of standard dressings in gastrointestinal surgery: A systematic review and meta‐analysis for guideline revision by the Japanese Society for Surgical Infection
Source: Ann Gastroenterol Surg. 2025 Jan 8;9(3):408–17. doi: 10.1002/ags3.12909 (PMC12080198; doi:10.1002/ags3.12909)

**a**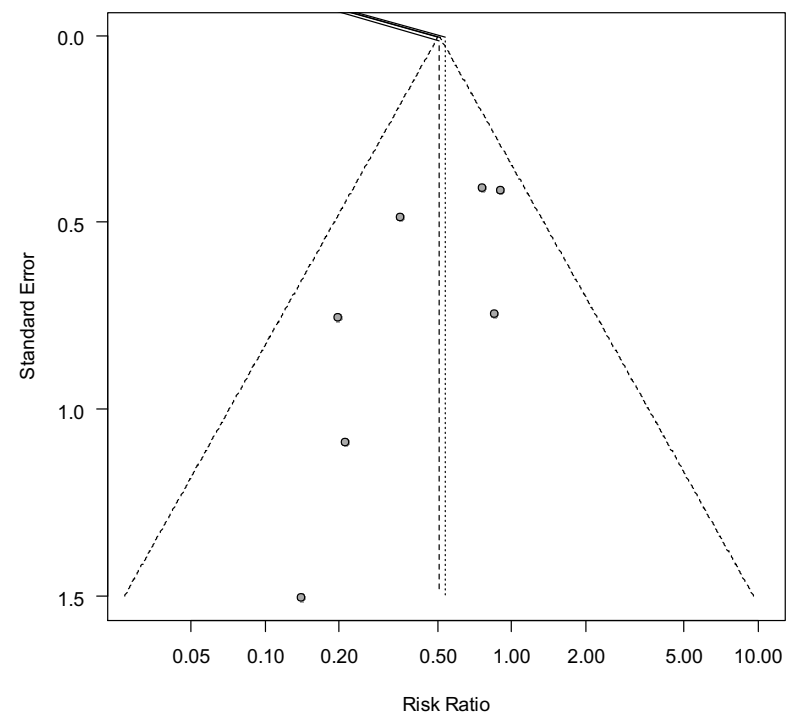**b**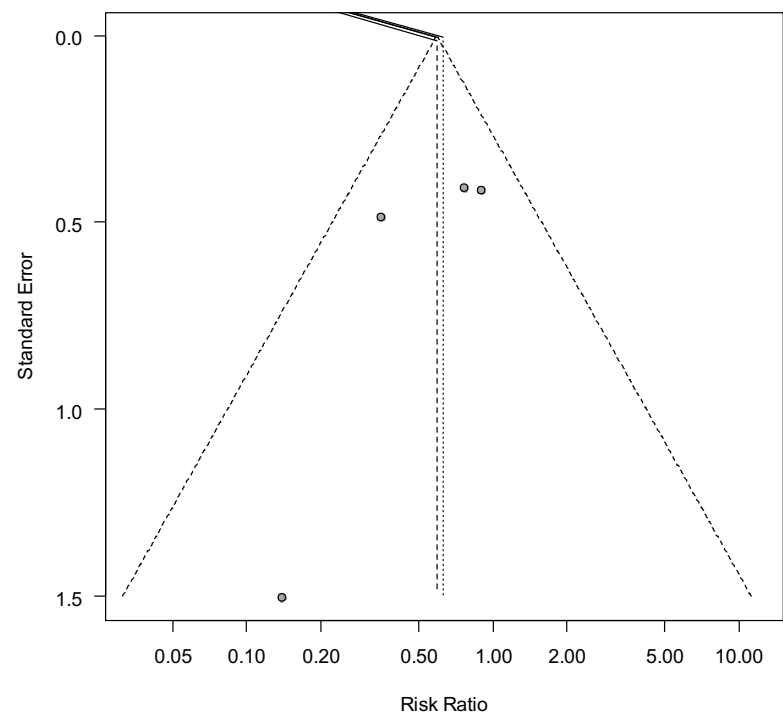**c**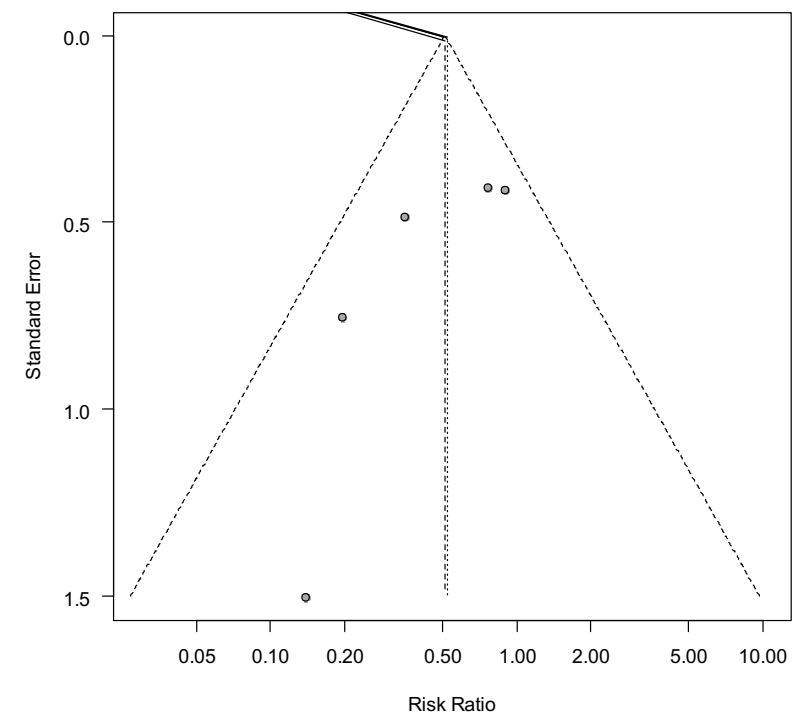

Supplement: Supplementary file 1 — Figure S1: The symmetrical funnel plots of meta‐analysis (a) comparing the incidence of surgical site infection (SSI) between any advanced dressings and standard dressings, (b) comparing the incidence of SSI between silver‐impregnated dressings and standard dressings, (c) comparing the incidence of SSI between any advanced dressings and standard dressings, specifically limited to colorectal surgery. All funnel plots suggest reporting bias with a paucity of studies with negative results. [file AGS3-9-408-s003.pdf]
