# Supplementary material for: Effectiveness of advanced dressings in preventing surgical site infections compared to that of standard dressings in gastrointestinal surgery: A systematic review and meta‐analysis for guideline revision by the Japanese Society for Surgical Infection
Source: Ann Gastroenterol Surg. 2025 Jan 8;9(3):408–17. doi: 10.1002/ags3.12909 (PMC12080198; doi:10.1002/ags3.12909)
Supplement: Supplementary file 3 — Data S2: Supplementary Material 2: Search strategy in Medline (through PubMed). [file AGS3-9-408-s002.docx]

Search strategy in Medline (through PubMed)

((Aquacel Ag Surgical[tiab] OR "carboxymethylcellulose sodium"[MeSH] OR "carboxymethylcellulose sodium"[tiab] OR "silver-containing"[tiab] OR silver*[mesh] OR silver*[tiab] OR aquacel*[tiab] OR Clearhesive[tiab] OR Bandages, Hydrocolloid[Mesh] OR hydrocolloid[tiab] OR Karayahesive[tiab] OR mepilex*[tiab] OR Silicon[Mesh] OR Silicon[tiab] OR waterproof[tiab] OR bacteria-proof[tiab]) AND ((Bandages[Mesh] OR "Occlusive Dressings"[Mesh] OR dressing*[tiab] OR bandag*[tiab])) AND ("Surgical Wound Infection"[Mesh] OR surgical site infection*[tiab] OR SSI[tiab] OR SSIs[tiab] OR surgical wound infection*[tiab] OR surgical infection*[tiab] OR post-operative wound infection*[tiab] OR postoperative wound infection*[tiab]))
